# Public Awareness and Knowledge of Neglected Tropical Diseases (NTDs) Control Activities in Abuja, Nigeria

**DOI:** 10.1371/journal.pntd.0003209

**Published:** 2014-09-25

**Authors:** Olatunwa J. Olamiju, Francisca O. Olamiju, Adebiyi A. Adeniran, Ifeanyi C. Mba, Chidera C. Ukwunna, Chukwu Okoronkwo, Uwem F. Ekpo

**Affiliations:** 1 Department of Research and Development, Mission to Save the Helpless (MITOSATH), Jos, Nigeria; 2 Department of Biological Sciences, Federal University of Agriculture Abeokuta, Abeokuta, Nigeria; 3 Department of Public Health, Federal Ministry of Health, Abuja, Nigeria; Kwame Nkrumah University of Science and Technology (KNUST) School of Medical Sciences, Ghana

## Abstract

The need to engage the public in Neglected Tropical Diseases (NTDs) control activities has become imperative in the context of morbidity reduction through preventive chemotherapy and community participation. Therefore, a survey was conducted among the general public to assess their knowledge and awareness of NTDs control activities in Nigeria. A simple questionnaire was administered to the general public attending a job fair in Abuja, Nigeria. Of the 461 respondents, a significant proportion 337 (73.1%) have heard of NTD before, but only 291 (63.1%) have good knowledge about NTDs. However, among the specific NTDs, only the control of onchocerciasis (50.8%) was of average public awareness in Nigeria, while all the other NTDs control activities were significantly less known to the general public. 397 (87.1%) stated that government support for NTD control activities is poor and were willing to assist to advocate for NTDs control. This survey demonstrates that despite government's numerous activities towards the control of NTDs in Nigeria, there is little sensitization of the general public. There is a need for policy changes that would raise the participation and involvement of the general public in NTDs control activities for sustainability.

## Author Summary

Nigeria carries the highest burden of NTDs in sub-Saharan Africa. However, public awareness and participation in NTD control activities is uncertain. The aim of this study was to provide information on the public perception of NTDs control activities in the context of preventive chemotherapy and morbidity reduction. We surveyed participants attending a job fair in Abuja, Nigeria in 2013 to find out their knowledge of NTDs control activities. Our results show that except for onchocerciasis control activities, many of the NTDs control activities currently ongoing in the country with national and international support are unknown to the public. There is a need for more efforts in publicizing NTDs control activities in the context of community participation.

## Introduction

Neglected Tropical Diseases (NTDs) are a group of disabling, chronic and disfiguring conditions that occur mostly in settings of extreme poverty, especially among poor rural and some disadvantaged urban populations [1]. NTDs cause substantial health and economic burden on poor populations in Africa, Asia, and Latin America [2] with about 534 000 deaths every year [1], and has similar disease burden to either malaria or tuberculosis [1], [3].

In sub-Saharan Africa, the diseases are the most common conditions affecting the poorest 500 million people living in the region and NTDs collectively produce a burden that may be equivalent to up to one-half of the malaria disease burden and more than double that caused by tuberculosis in sub-Saharan Africa [3].

There are seventeen NTDs that have being identified by WHO to be of particular importance due to their frequency among poor communities and their clinical, social and economic impact [4]. These include soil transmitted helminthiasis (STH) that comprises of ascariasis, trichuriasis and hookworm infections; onchocerciasis, schistosomiasis, lymphatic filariasis (LF), human African trypanosomiasis (HAT), chagas disease, echinococcosis, leishmaniasis, dracunculiasis, buruli ulcer, leprosy, trachoma, dengue fever, rabies, cysticercosis/taeniasis, food borne trematodiases and yaws [4]. Among these NTDs, Nigeria has the greatest number of people infected or at risk with schistosomiasis (29 million), lymphatic filariasis (80–121 million at risk), ascariasis (55 million), hookworm (38 million) and Trichuriasis (34 million) among all the African nations [3], [5], [6].

The control of NTDs in many sub-Saharan African countries has been made less important by other health priorities with the highest priority given to HIV/AIDS, malaria and tuberculosis [7] even though the resources required to effectively control the NTDs are relatively low compared to what have already been spent to fight AIDS, tuberculosis and malaria [8]. Control activities targeted at several NTDs have been ongoing in the country since 1980s, through internationally supported national control programmes. These control activities were for dracunculiasis, onchocerciasis, lymphatic filariasis, schistosomiasis, leprosy, trachoma and soil transmitted helminthiasis [6]. Though there is some funding for these control activities by both the federal and state governments the level has been relatively low [6], [9]. However, given the need for public participation in these controls activities and its sustenance, particularly those NTDs that can be controlled through preventive chemotherapy drug; there is therefore the need to perceive public and policy makers' knowledge about NTDs and its control activities in Nigeria.

## Methods

### Study site

The study was carried out in Abuja, the Federal Capital Territory of Nigeria. Abuja is located between latitude 8.25 and 9.20 north of the equator and longitude 6.45 and 7.39 east of Greenwich Meridian. Abuja is geographically located in the centre of the country and has a landmass of approximately 7,315 km^2^. The city is well planned and developed and is home to Nigerian seeking employment or business opportunities since it is the seat of the federal government. Therefore, Nigerian from all parts of the country troops into Abuja daily either for business, job opportunities or for settlement.

### Study design

The study is cross-sectional using a questionnaire designed to test public knowledge and awareness of NTDs control activities in Nigeria. The authors used a well-advertised job fair which was attended by governmental officials, business owners, policy makers, politicians, students, job seekers and the general public. Participants were randomly approached by the interviewers, asked to indicate interest in filling the questionnaire, but without devolving personal information. Participants, who offered to complete the questionnaire where then, requested to complete an informed consent form first, after the purpose of the study had been thoroughly explained to them.

### Ethical approval

The ethical approval for the study was obtained from the Ethics Review Committees of the Federal University of Agriculture, Ogun state and Federal Ministry of Health, Abuja.

### Sampling selection

Sampling was done randomly and effort was made to reach all participants that attended the conference except those that opted out. Caution was also taken not to allow the questionnaire completion affect participants' participation at the conference. Participants were asked to pick from rolled pieces of paper wrapped “yes” and “no”. Only participants that picked “yes” were allowed to complete a questionnaire after completing a consent form.

### Questionnaire administration

An interviewer-administered questionnaire was used for the survey. A pre-implementation meeting was held during which the questionnaire as well as possible responses to the questions was reviewed among six trained interviewers. The questionnaire was field tested on few people in Abuja that were not attending the job fair and an appraisal conducted and ambiguous questions were amended. Interviewers were assigned to different sections of the fair to avoid bias. The survey was carried out in August 2013 during a 3-day job fair at Abuja, Federal Capital Territory.

### Data management and data analysis

Data from questionnaires were entered and analysed using Statistical Package for Social Science (SPSS) version 16 for Windows. Descriptive statistics was used to categorize important variables and chi-square was used to test for significance. Level of significance was set at 95%.

## Results

A total of 461 job fair participants completed questionnaires for the study and this comprised 244 (58.2%) males and 175 (41.8%) females with age range from 18–48 years and mean age of 27.8±0.185 years. 75.3% of the respondents were job seekers (Table 1).

**Table 1:** Demographic characteristics of respondents.

	Number of respondents	Percentage (%)
**Sex**		
Male	244	52.9
Female	175	38.0
No sex information	42	9.1
**Occupation**		
Job seekers	347	75.3
Private	35	7.6
Civil servants	5	1.1
Government official/Policy maker	38	8.2
Media	5	1.1
Public servants	18	3.9
Academic	13	2.8
**Age-group**		
15–20	4	0.9
21–25	141	30.6
26–30	216	46.9
31–35	44	9.5
>35	13	2.8
No Age information	43	9.3

Majority of the respondents have a good knowledge about the meaning of NTDs as 63.1% correctly got the full meaning of NTDs and 62% choose onchocerciasis as example of NTD that they are aware of its control. 73.1% of the respondents also claimed to have heard of the presence of the NTDs in Nigeria and 40.4% stated electronic media as the source of awareness (Table 2).

**Table 2:** General knowledge of the public about NTDs.

	Number	Percentage
**Do you know the full meaning of NTD**		
Yes	291	63.1
No	170	36.9
**Have you heard of any NTD in Nigeria**		
Yes	337	73.1
No	124	26.9
**If yes, where did you hear of NTD**		
Scientific Journal	81	22.6
TV/Radio/Electronic media	142	39.6
Conferences/meetings	39	10.9
All of the above	97	27
**Is NTD a problem of public Health importance in Nigeria?**		
Yes	428	92.8
No	33	7.2
**If yes, why do you think so?**		
I read about it in the dailies	88	20
I read about it in scientific journal or event	119	27
I know about people suffering from the disease	154	35
I am not sure	52	11.8
I come from an endemic area	27	6.1
**Do you or have you seen anyone been affected by NTD before?**		
Yes	262	56.8
No	199	43.2
**Among these diseases, which can be consider as a NTD?**		
Onchocerciasis/river blindness	286	62.0
Malaria	60	13.0
HIV/AIDS	18	3.9
All of the above	97	21.0

Approximately 93% of the respondents agreed that the diseases are of public health importance in Nigeria with 35% confirming knowing people that are suffering from one of the diseases as the evidence; 27% claimed to have read about them in scientific journal or event and 20% of the respondents claimed reading about them in the dailies. Further analysis of those that claimed to have read about NTD as a public health problem in scientific journals showed 72.3% as professionals and only 27.7% were job seekers.

Onchocerciasis was the disease respondents showed the highest knowledge about with 50.8% of respondents having knowledge of its control activities. This is followed by lymphatic filariasis with 25.2%; leprosy (24.7%), trachoma (22.1%) HAT (21.9%); schistosomiasis (21.5%) and STH infections (21.3%) (Table 3). Diseases which respondents showed limited awareness about include leishmaniasis (6.1%), Buruli ulcer (6.9%), Chagas diseases (8.5%), rabies (14.8%) and dracunculiasis (16.9%). 55% of the males have heard about one NTD control activity or another and although 1.519 (0.996–2.317 95% CI) more likely to have heard about an NTD control activity than the females, there is no statistically significant difference between the group. Also, 50.9% of those that have heard of an NTD control activity are between the age group 26–30 years and there is no significant difference among the age groups (Table 4).

**Table 3:** Knowledge of respondents about specific NTDs control activities.

NTDs	Number of respondents	Percentage
STH^a^	98	21.3
Schistosomiasis^a^	99	21.5
Onchocerciasis^b^	234	50.8
Leprosy^c^	114	24.7
Trachoma^d^	102	22.1
Buruli ulcer^h^	32	6.9
Leishmaniasis^h^	28	6.1
Rabies^h^	68	14.8
Human African Trypanosomiasis^e^	101	21.9
Lymphatic filariasis^f^	116	25.2
Dracunculiasis^g^	78	16.9
Chagas diseases^h^	39	8.5
Any NTD control activities	300	65.1

a National Schistosmiasis/STH Control Program.

b National Onchocerciasis Control Program.

c National Tuberculosis and Leprosy Control Program.

d National Eye Health Program.

e Pan African Tse-tse and Trypanosomiasis Eradication Campaign.

f Lymphatic Filariasis Elimination Program.

g Guinnea worm Eradication Program.

h No control program.

**Table 4:** Knowledge of any NTD control activities by sex and age.

Respondents that have heard of any control activities	Yes	No	p-value	Odd ratio	95%CI
**Sex**					
Male	155 (55)	61 (65)	0.052	1.519	0.996–2.317
Female	127 (45)	43 (35)			
Total	282	104			
**Age group**					
15–20	3 (1.1)	1 (0.7)	0.125		
21–25	104 (37.0)	42 (27)			
26–30	143 (50.9)	47 (53.3)			
31–35	24 (8.5)	8 (14.6)			
>35	7 (2.5)	3 (4.4)			
Total	281	101			

A high proportion of 87.1% of the respondents were of the opinion that government awareness and responses to NTDs was poor. Also, 92.2% of the respondents were willing to participate in NTD related activities in their zones with 63.3% of the opinion that advocacy, more funding and research are necessary to reduce the burden of NTDs in Nigeria. The most activities respondents will like to partake in are advocacy with 51.4% of respondents showing interest (Table 5).

**Table 5:** Different opinions of the public about NTDs control activities.

	Number	Percentage
**What do you think can be done to reduce the burden of NTD in Nigeria**		
More research	81	17.6
Advocacy	60	13.
More Funding	28	6.1
All of the above	292	63.3
**Do you think there is enough awareness about NTD among government institution/policy makers and MDA?**		
Yes	59	12.9
No	397	87.1
**Would you be will to participate in any activities related to NTD in your zone/state?**		
Yes	425	92.2
No	36	7.8
**If yes, Please tick your preference activities**		
Advocacy	222	51.4
Fund raising	21	4.9
Networking	113	26.2
Legislation	24	5.6
Others	52	12.0

## Discussion

Public awareness and perception of a disease are important factors in shaping the necessary policies toward the control of such diseases. In the context of NTDs control, public knowledge may influence policy formulation and implementation. Although this study shows that generally many respondents have heard about NTDs control activities in Nigeria, however, their knowledge on specific NTD control activities is low relative to the percentage of the respondents that considered the diseases as a public health problem in Nigeria. It was also interesting to know that only 63.1% know the full meaning of the NTDs acronym. This finding shows that public awareness of NTD control activities is low in the surveyed population, which was visitors of a trade fair in Abuja, the Nigeria Federal capital city. This finding is in support of other studies in Nigeria [10], [11] and many African countries [12]. However, there are instances where there is high level of awareness of the diseases but the awareness of control activities is low [13], [14].

Currently, onchocerciasis appears to be the NTD that the public seems to be mostly aware of its control activities. This may be due to the fact that onchocerciasis control activities in Nigeria has been ongoing since 1991 [15] and there are several national and international partners such as MITOSATH, UNICEF, WHO and Sight savers, working on onchocerciasis control in Nigeria which are visible to the general public. In addition, the availability of the popular drug Ivermectin which is distributed freely to population at risk using the program that involves community members; Community Directed Treatment with Ivermectin (CDTI) has also contributed to making onchocerciasis control activities a public knowledge in Nigeria [15]. Lymphatic filariasis control which is also linked to onchocerciasis control through integrated treatment was the second due to relative newness of LF control. Despite the availability of Preventive Chemotherapy Drug for schistosomiasis and soil transmitted helminthiasis which also carries heavy burden in Nigeria; the awareness about their control activities was low. This could be due to poor government funding of schistosomiasis and soil transmitted helminthiasis control programme for long time [3]. However, leprosy, soil-transmitted helminth infections, lymphatic filariasis, onchocerciasis and Human African trypanosomiasis have been shown to be better known by communities than the rest of the NTDs [12].

Respondents' erroneous knowledge about control activities for Buruli ulcer, leishmaniasis, rabies and chagas diseases could have been negatively influenced by knowledge of people suffering from these diseases as 35% of respondents (Table 2) claimed to know of people suffering from one NTD or another and there are no specific control program or control activities for these diseases (Table 3) even though there have been reported cases in Nigeria. This suggests that people knowledge about control activities could be relatively influenced by knowledge of infected person.

The knowledge about NTD control activities is higher in males than the females although not statistically significant (Table 4) and agrees with report from Cameroon that males showed more knowledge about NTDs than females [12].

It is alarming to know that the general public believes that government has done little about NTDs control in spite of NTD targeted control programmes, this could be due to poor funding of these programmes by the government when compared to other control program like malaria and HIV, which suggests the need to engage more stakeholders and provide more enlightenment on government activities on specific NTDs. This give further credence to the fact that NTDs controls are given lesser priorities compared to HIV/AIDS, malaria and tuberculosis control in sub-Saharan Africa [7]. The willingness of respondents to participate in advocacy for NTDs activities, is an opportunities for the Nigerian government and its global partners in NTD control to diverse ways to increase public participation in NTDs control activities. Resources and funds should be allocated to public awareness programmes, jingles in electronic media, use of mobile telephony to send information as it is being done for malaria control. Without the public being mobilised to participate in NTD control activities in Nigeria, it would be difficult and not impossible for the current intervention using preventive chemotherapy drug to succeed.

Although the size and non-representative of the population sampled posed a major limitation; the study however is preliminary and the outcome will provide a baseline from which more in depth study that would encompass different segment of the society can be carried out.

### Conclusions

Public awareness and participation in NTDs control activities in Nigeria, especially in the targeted population of our survey, is low and this poses a serious threat to the control of NTDs as individuals' lack of the basic knowledge about these diseases including the causative organism and transmission, would continue to impede control activities. Therefore public participation to discuss on the high burden NTDs is suggested to increase the public awareness and thus facilitate support for control activities.

## Supporting Information

Checklist S1 STROBE checklist.
(DOC)

Questionnaire S1 Sample question on public awareness and knowledge survey on NTDs control activities in Nigeria.
(DOC)
