# Supplementary material for: Public Awareness and Knowledge of Neglected Tropical Diseases (NTDs) Control Activities in Abuja, Nigeria
Source: PLoS Negl Trop Dis. 2014 Sep 25;8(9):e3209. doi: 10.1371/journal.pntd.0003209 (PMC4177755; doi:10.1371/journal.pntd.0003209)
Supplement: Questionnaire S1 — Sample question on public awareness and knowledge survey on NTDs control activities in Nigeria. (DOC) [file pntd.0003209.s002.doc]

**SAMPLE QUESTIONNAIRE ON PUBLIC AWARENESS AND KNOWLEDGE SURVEY ON NTDs CONTROL ACTIVITIES IN NIGERIA**

1. **Do you know the full meaning of the acronym NTD**
2. Natural Tropical Diseases
3. Neutral Tropical Diseases
4. Neglected Tropical Diseases
5. Native Tropical Diseases
6. **Among these diseases, which can be consider as a NTD**
7. Onchocerciasis/river blindness
8. Malaria
9. HIV/AIDS
10. All of the above
11. **Have you heard of any NTD in Nigeria**

Yes No

1. **If yes, where did you hear of NTD**
2. Scientific Journal
3. TV/Radio/Electronic media
4. Conferences/meetings
5. All of the above
6. **Is NTD a problem of public Health importance in Nigeria**

Yes No

1. **If yes why do you think so**
2. I read about it in the dailies
3. I read about it in scientific journal or event
4. I know about people suffering from the disease
5. I am just guessing
6. I come from an endemic area
7. **Do you or have you seen anyone been affected by NTD before**

Yes No

1. **What do you think can be done to reduce the burden of NTD in Nigeria**
2. More research
3. Advocacy
4. More funding
5. All of the above
6. **Would you be will to participate in any activities related to NTD in your zone/state?**

Yes No

1. **If yes, Please tick your preference activities**
2. Advocacy
3. Fund raising
4. Networking
5. Legislation
6. Others
7. **If no, why (tick as appropriate)**
8. I do not have the time
9. NTD is not that important/not serious in my zone/state
10. I do not have the training
11. I need to know more about NTD to participate
12. **Do you think there is enough awareness about NTD among governments institution/policy makers and MDA? From what you have said so far?**

Yes No

**13.** **List all the NTDs you know about their control activity:**

___________________________________________________________________

___________________________________________________________________

___________________________________________________________________

___________________________________________________________________

___________________________________________________________________

___________________________________________________________________

___________________________________________________________________

___________________________________________________________________

___________________________________________________________________

___________________________________________________________________

___________________________________________________________________

___________________________________________________________________

___________________________________________________________________

___________________________________________________________________

___________________________________________________________________

___________________________________________________________________

**Name** ________________________**OCCUPATION** _______________________

**Telephone_____________________Sex_____________ Age______________**
